# Characterization of Full‐Length Antibody Complexes Through HDX‐Mass Spectrometry Coupled With NMR Spectroscopy: New Insight Into Avelumab/PD‐L1 Interaction

**DOI:** 10.1002/anie.1540355

**Published:** 2026-08-22

**Authors:** Francesca Sacco, Fabio Baroni, Angela Capolupo, Sofia Petrocchi, Jonathan Zöller, Linda Cerofolini, Pasquale Russomanno, Rebecca Calamandrei, Bianca Susini, Siyu Lin, Fabio D'Amici, Enrico Ravera, Julian Langer, Marco Fragai

**Affiliations:** ^1^ CERM‐CIRMMP University of Florence Sesto Fiorentino Italy; ^2^ Analytical Development & Innovation, NBE, EMD Serono Inc Guidonia Rome Italy; ^3^ Max Planck Institute of Biophysics Frankfurt am Main Germany; ^4^ Department of Chemistry (DICUS) University of Florence Sesto Fiorentino Italy; ^5^ Max Planck Institute For Brain Research Frankfurt am Main Germany

**Keywords:** antibodies, biotherapeutics, immune checkpoints, mass spectrometry, NMR spectroscopy

## Abstract

Recombinant biomacromolecules have significantly transformed pharmaceuticals, in particular monoclonal antibodies (mAbs) that target key areas in oncology, immunology, inflammation, and neurodegenerative diseases. Developing biotherapeutics requires a detailed understanding of their biological properties. This, in turn, brings about the importance of characterizing higher‐order protein structures at each stage, from production to patient. The flexibility and size of the mAbs often prevent achieving atomic‐level structural information in the pharmaceutical formulation by traditional methods. In this study, we introduce an integration of solid‐state nuclear magnetic resonance (SSNMR) and hydrogen–deuterium exchange mass spectrometry (HDX‐MS) to explore the interaction between Avelumab and the programmed cell death‐ligand 1 (PD‐L1). With this approach, we were able to obtain epitope (by both NMR and HDX) and paratope mapping (by HDX), together with a detailed characterization of the structural rearrangements arising upon binding. This methodology provides both mechanistic and structural insights important for the design and optimization of biotherapeutics.

## Graphical abstract

Combining HDX‐MS and NMR enables the mapping of epitopes and paratopes in full‐length antibody complexes, revealing transient interactions and potential artifacts.

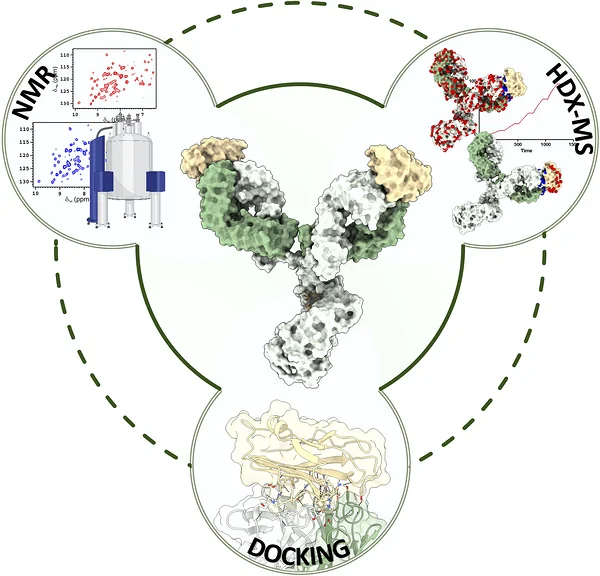

## 1 Introduction

Antibodies, also known as immunoglobulins, are crucial components of the immune system, playing a critical role in defending the organism against pathogens such as bacteria, viruses, and other harmful substances. They are highly versatile and find a wide range of applications beyond their natural immune function [1]. In medicine, antibodies are extensively employed as therapeutic agents for treating various diseases, including cancer, autoimmune disorders, and infectious diseases. Additionally, they have proven to be essential tools in diagnostics, research, and biotechnology, enabling precise detection, targeting, and manipulation of specific molecules or cells [2, 3]. Therefore, understanding the precise interactions between antibodies and their target pathogens or antigens is crucial for designing and improving antibodies with optimal binding affinity and specificity. Hence, epitope mapping is essential and provides valuable insights for the development of therapeutic antibodies: (i) revealing how candidate antibodies bind to a target to enable lead candidate selection, (ii) understanding the binding mechanism for optimization and design of novel bispecific proteins and antibody‐drug conjugates, and (iii) monitoring the higher‐order structure (HOS) of mAbs during pharmaceutical formulation development and manufacturing [4, 5]. Several biophysical methods can be used to map epitopes in these complexes, including x‐ray crystallography [6, 7, 8, 9], nuclear magnetic resonance [10], cryo‐electron microscopy [11, 12, 13], and mass spectrometry [14, 15]. Each method has its own strengths and weaknesses [16, 17], and it is becoming increasingly apparent that the combination of orthogonal analytical techniques is crucial to compensate for the inherent limitations of each individual technique, ensuring robustness and providing cross‐validation of the results [18, 19, 20, 21, 22]. In particular, antibodies are large but very flexible macromolecules, and these features prevent the acquisition of high‐resolution structures of their complexes with target proteins. Therefore, these studies typically rely upon fragments of the selected antibodies (such as single‐chain variable fragments, or scFvs) in complex with their targets [23, 24]. While this information is crucial, it often overlooks allosteric effects and the potential influences of other domains or functional groups that are present in full‐length antibodies or fusion proteins. In this scenario, developing strategies to study intact large proteins, such as monoclonal antibodies (mAbs) or fusion proteins, using high‐resolution techniques has become highly important in biotech development. Over the last decade, solid‐state NMR has emerged as a powerful technique for the characterization of large protein assemblies and protein–ligand complexes, providing atomic‐level information [10, 25, 26, 27, 28, 29, 30, 31, 32, 33, 34, 35, 36, 37, 38, 39, 40, 41, 42, 43, 44, 45, 46, 47, 48, 49, 50]. More recently, this technique has demonstrated an outstanding capability in providing atomic‐level epitope mapping of whole antibodies and fusion proteins derived from pharmaceutical formulations [10]. Although experimentally independent modeling programs are available [51, 52, 53], structural data from biophysical measurements remain essential for validating the resulting biomolecular complexes. In this regard, structural data can be integrated into information‐driven flexible docking programs for the modeling of biomolecular complexes (e.g., Haddock [54]). Since therapeutic antibodies are available only at natural isotopic abundance and isotopic enrichment remains technically challenging, SSNMR alone provides experimental information on the epitope, but not on the paratope. Consequently, a comprehensive understanding of the mAb‐target complex, starting from the pharmaceutical formulation, is still lacking. This limitation may be addressed by (i) computational prediction of mAb interacting regions (e.g., SAbPred [55], proABC‐2 [56]), (ii) available x‐ray data on mAb fragments, (iii) future breakthroughs in expressing isotopically enriched antibodies in mammalian cells, or (iv) by the integration of another high‐resolution biophysical technique, such as hydrogen–deuterium exchange mass spectrometry (HDX‐MS). In particular, HDX‐MS has been widely used in epitope mapping and ligand binding characterization [57, 58, 59]. HDX‐MS measures the deuterium uptake differences of the target protein and allows the investigation of binding sites on the epitope and paratope and conformational changes upon binding. Although HDX‐MS lacks the residue‐level resolution of SSNMR, it provides complementary peptide‐level information on conformational dynamics and solvent accessibility in both the antibody and the target protein.

Therefore, combining HDX‐MS and SSNMR can be a powerful approach for studying antigen–antibody interactions to map both epitopes and paratopes. This integrated approach can offer insights into conformational dynamics, protein folding/unfolding mechanisms, binding interfaces, and allosteric effects. The feasibility and advantages of this strategy have been investigated by analyzing the interaction of Avelumab with the N‐terminal domain of the programmed cell death‐ligand 1 (PD‐L1). Avelumab is a mAb directed against PD‐L1, whose expression is often upregulated in various cancers in order to evade immune surveillance [60, 61]. The programmed cell death‐protein 1/programmed cell death‐ligand 1 (PD‐1/PD‐L1) axis serves as an immune checkpoint mechanism that, under normal circumstances, fosters self‐tolerance and shields the host from autoimmune reactions. For its key role in immune system regulation, the ectodomain of PD‐L1 serves as a focal point for numerous antibodies currently in use or under development for treating cancers with high levels of this protein [62, 63]. Considering the therapeutic relevance, the Avelumab/PD‐L1 immune checkpoint blockade is being actively investigated. The structural elements of Avelumab are schematically represented in Figure 1. As for all mAbs, target binding is achieved by the means of complementarity‐determining regions (CDRs), located on the heavy and light chains of the antigen‐binding fragment (Fab). The CDR loops of Avelumab target the β−platform of PD‐L1 constituted by the C, C′, F, and G strands and CC′ loop (Figure 1).

**FIGURE 1:**
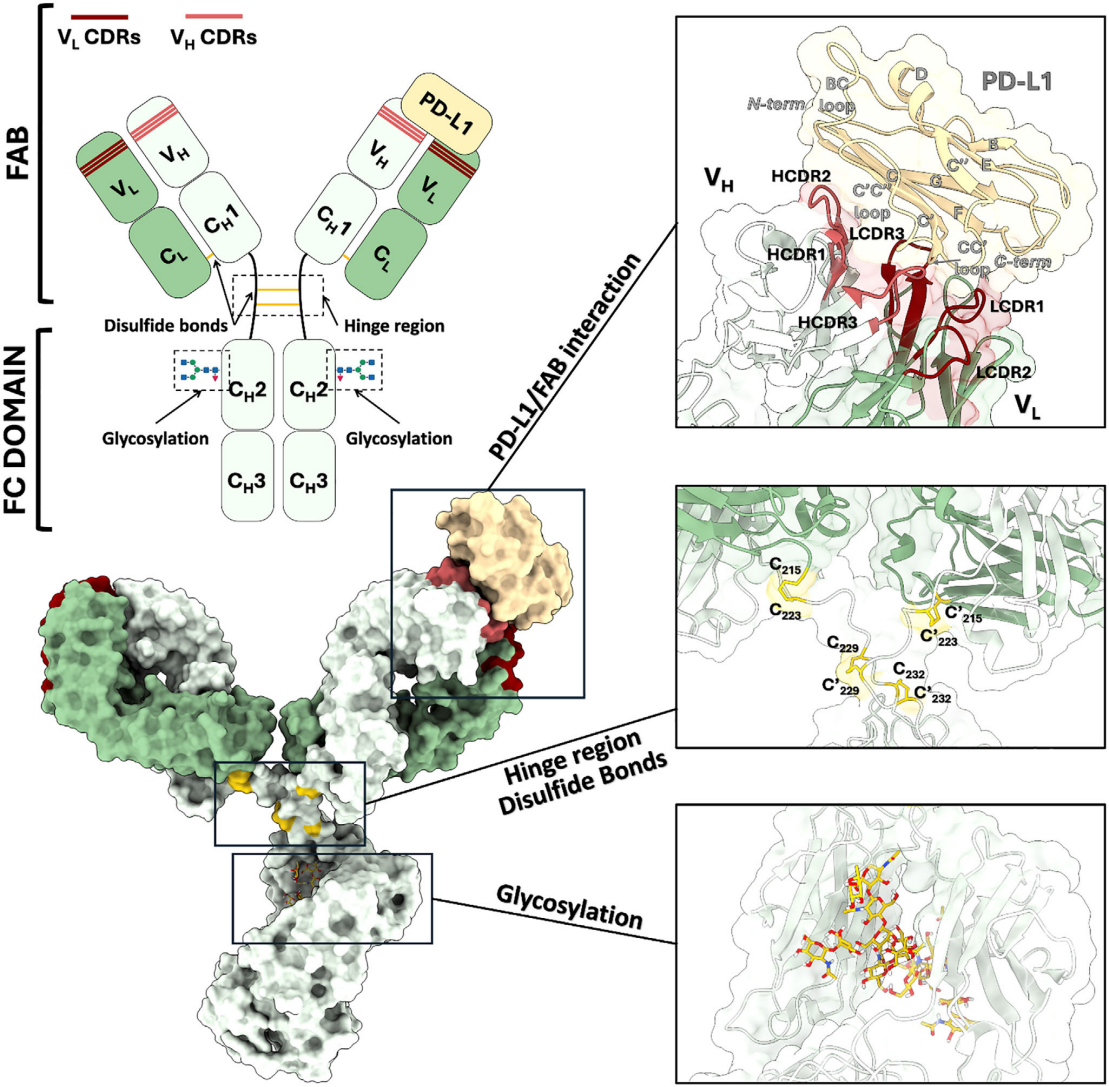
Schematic representation of an IgG1 antibody (e.g., Avelumab). Light and heavy chains are colored in dark and pale green, respectively. The Fab domain, consisting of the variable regions (*V*_H_ and *V*_L_) and the constant regions (C_H_1 and C_L_), is shown to be binding to the antigen (PD‐L1, PDB: 5GRJ) with the CDRs highlighted in red. The crystallizable fragment (Fc) and key structural features of antibodies are also highlighted. In addition to the primary Fab‐related activity, the Fc mediates the effector functions by binding to Fc gamma receptors (FcγR) and Fc receptors (FcRn). The binding with the first one induces an adaptive immune response and antibody‐dependent cellular cytotoxicity (ADCC); the latter—at the interface between C_H_2 and C_H_3 domains—regulates the pharmacokinetics and clearance of antibodies [64]. The disulfide bonds and the glycosylation site (N300) are also shown in enlarged views. The structural elements of PD‐L1 are indicated in the upper enlarged panel with gray labels.

In our previous study [10], we characterized the epitope of the isotopically enriched PD‐L1 ectodomain complexed with an anti‐PD‐L1 full‐length fusion protein with SSNMR. The fusion protein (a product in development by Merck KGaA) was obtained by covalently linking Avelumab (see Figure 1) to two identical extracellular domain proteins capable of binding another therapeutic target. In particular, Avelumab shares the same primary structure as the fusion protein, except for three amino acid substitutions in regions not involved in PD‐L1 binding. However, the information related to the paratope mapping on the fusion protein was missing. In this study, we examine the effects induced by the binding in both partners, Avelumab (EMD Serono and Pfizer) and PD‐L1, using HDX‐MS and SSNMR. This approach offers insights into the conformational changes of antibodies, which can help in further optimizing and maturing antibodies during biotherapeutic development.

## 2 Results and Discussion

### 2.1 Paratope Mapping by HDX‐MS

The binding affinity of Avelumab for the target PD‐L1, in the chosen experimental conditions, was first assessed using surface plasmon resonance (see Figure S1 and Table S1), which revealed an equilibrium dissociation constant in the picomolar range and a slow dissociation rate. HDX‐MS was then utilized to examine the effects and conformational changes occurring in Avelumab upon binding to PD‐L1.

After fine‐tuning the experimental conditions, a sequence coverage of 83.8% for the heavy chain (144 peptides, redundancy 4.12) and of 95.4% for the light chain (59 peptides, 3.03 redundancy), was achieved (Figures S2 and S3). All peptides displaying statistically significant differences in deuterium uptake between binding and control conditions were used for visualization on the Avelumab structure described by Saporiti et al. (Figure 2) [65].

**FIGURE 2:**
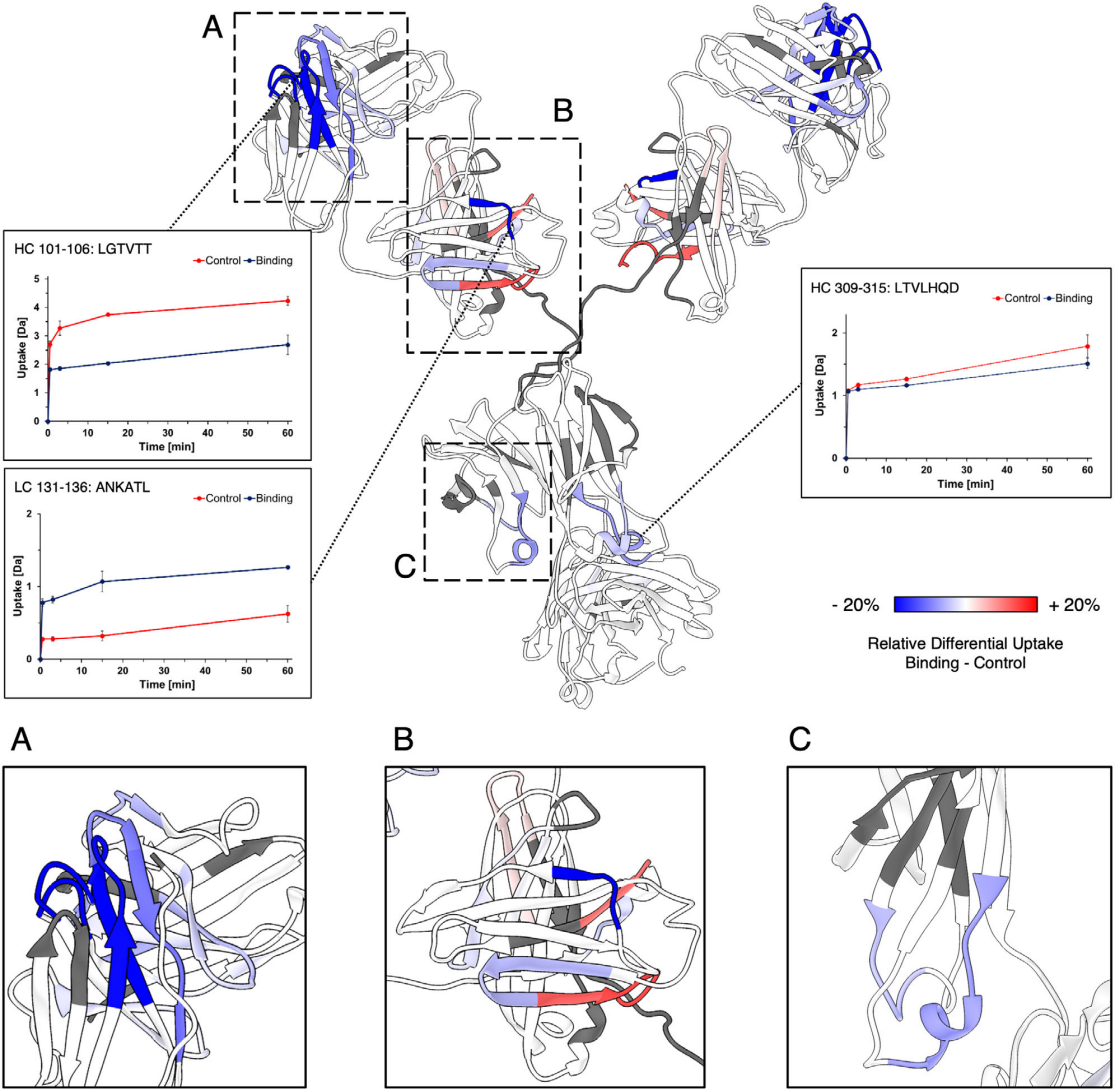
HDX results of Avelumab after PD‐L1 binding and 60 min of deuterium labelling. Deuterium uptake differences were plotted onto the model structure of Avelumab [65]. The uptake differences are indicated with a color gradient ranging from blue (decreased) to white (unchanged) to red (increased uptake). Non‐identified residues are colored in grey. Panels A, B, and C represent zoom‐in sections of the Fab domain, hinge regions, and Fc, respectively. Representative peptide uptake plots are shown with standard deviation error bars. The control condition is shown in red, and the binding condition in blue. Statistical significance of differences in deuterium uptake was assessed using a two‐sided t‐test; all time points shown indicate statistically significant differences.

Figures 3 and S4 provide a detailed view of the results obtained for the Fab region of the antibody. A decreased deuterium uptake can be caused by rigidification of the protein structure or by protection from a bound ligand. Here, this protection might be caused by PD‐L1 binding, indicating an involvement of the CDRs [7, 8]. The strongest effects were observed in segments located in the binding region of the Fab domain, which showed a marked decrease (48%–51%) in deuterium uptake upon binding (residues G102‐T106, CDR3‐V_H_). Peptides in the light chain (residues I20‐Y34, CDR1‐V_L_, and S95‐S97, CDR3‐V_L_) together with segments in the heavy chain (residues L101‐T106 and P53‐F59, CDR3‐V_H_ and CDR2‐V_H_, respectively) display a significant decrease in deuterium uptake.

**FIGURE 3:**
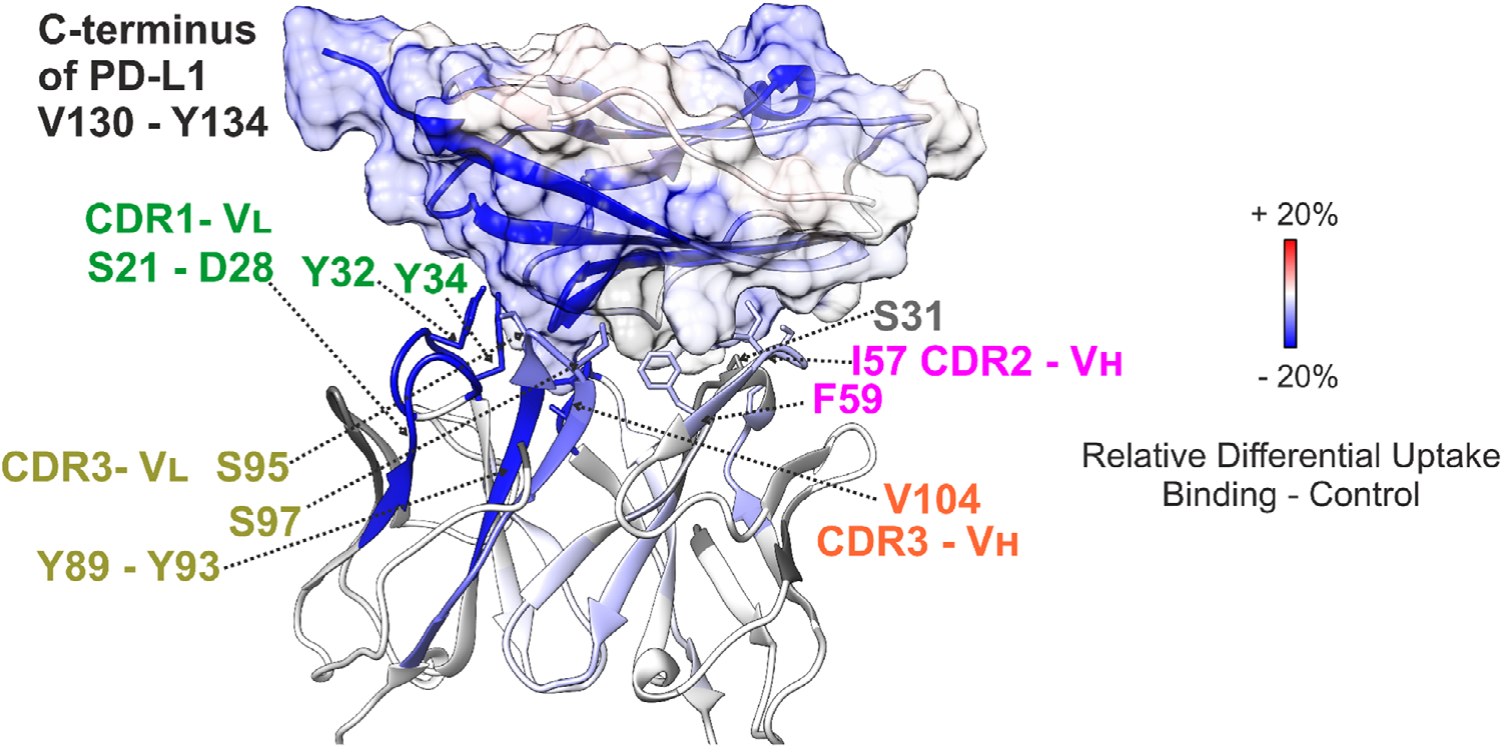
Close‐up view of the interaction of PD‐L1 and Avelumab. Selected residues in the binding epitope are shown as a stick representation (structure color scheme by differential deuterium uptake, as previously). The fragments identified by HDX‐MS analysis match the segments in the heavy and light chains of Avelumab contacting PD‐L1, as revealed by the x‐ray structure. Among those on the heavy chain, only serine 31 (in gray) was not covered by HDX. Residue labels of the Avelumab binding region are highlighted in different colors: green for residues in CDR1 of the light chain; olive for residues in CDR3 of the light chain; magenta for residues in CDR2 of the heavy chain; orange for residues in CDR3 of the heavy chain. Residues of the C‐terminus of PD‐L1 showing strong protection from deuterium exchange have been indicated in black in the figure.

Previous studies showed that changes in the constant domains (see Figure 1) are influenced by antigen binding, in a structural “cross‐talk” between the variable and constant domains [66, 67]. The increased deuterium uptake in segments E206‐S216 of the light chain and Y201‐K213 of the heavy chain (Figures 2B and S5) seems to support these findings. Indeed, these changes may be consistent with the conformational rearrangements occurring on the loop of the constant heavy 1 (C_H_1) domain. Such rearrangements have been proposed to play a role in the functional diversity of IgGs by altering the spatial relationship between the C_H_1 domain and the crystallizable fragment (Fc) [68, 69]. However, in our data, no peptides from the hinge region were detected, most likely due to reduced digestion efficiency associated with the proximity of the two disulfide bridges and the glycosylation site (N300).

In the Fc, multiple segments near the interface between the C_H_2–C_H_3 domain (residues L245–M255 and L309–D315 in the heavy chain) showed decreased deuterium uptake (Figures 2C and S6). This region (particularly residues H310, H435, and I253) is known to be involved in salt‐bridge interactions with FcRn receptors [70, 71]. We have correlated these allosteric modifications, which result in differential deuterium exchange, with the recently revealed long‐range structural rearrangements in the Fc domain. These structural rearrangements are relevant because they affect the affinity of FcγRs and FcRn for the Fc domain [72, 73]. The study by Sun et al. describes the changes in solvent‐accessible surface area (SASA) between the free and antigen‐bound states in both Fab and Fc regions of protein side chains. In particular, when studying the tertiary antigen‐IgG1–FcRn complex, they observed that residues K249–M255 show reduced solvent accessibility, which was correlated with Fab‐antigen binding, rather than with non‐specific interactions of the antigen in the Fc domain [73].

To better understand the results, we compared the HDX data with findings from Liu et al. [8], which analyzed the complex between PD‐L1 and the single‐chain variable fragment (scFV) of Avelumab using x‐ray crystallography (PDB code: 5GRJ). Our HDX results are consistent with the previously reported x‐ray data (Figure 4A,B), which identified the C, C′, F, and G strands and the CC′ loop of PD‐L1 as part of the binding epitope (Figure 1, enlarged view at the top). These regions are also protected in our experiments, supporting the same interaction interface, except for S31 on the heavy chain (not identified by HDX). Interestingly, we detected additional residues displaying decreased deuterium uptake upon binding of PD‐L1. In the light chain, the Y89‐Y93 and V100‐K106 segments show a decreased deuterium uptake upon PD‐L1 binding. In addition, residues S21‐D28, located in a region of the light chain that does not directly contact PD‐L1, exhibit a marked reduction in deuterium uptake, up to −21%. The bending of the C‐terminus of this PD‐L1 construct, which is known to be highly flexible [10], may reflect stabilization of this region, either directly or allosterically, through engaging additional contacts with the mAb.

**FIGURE 4:**
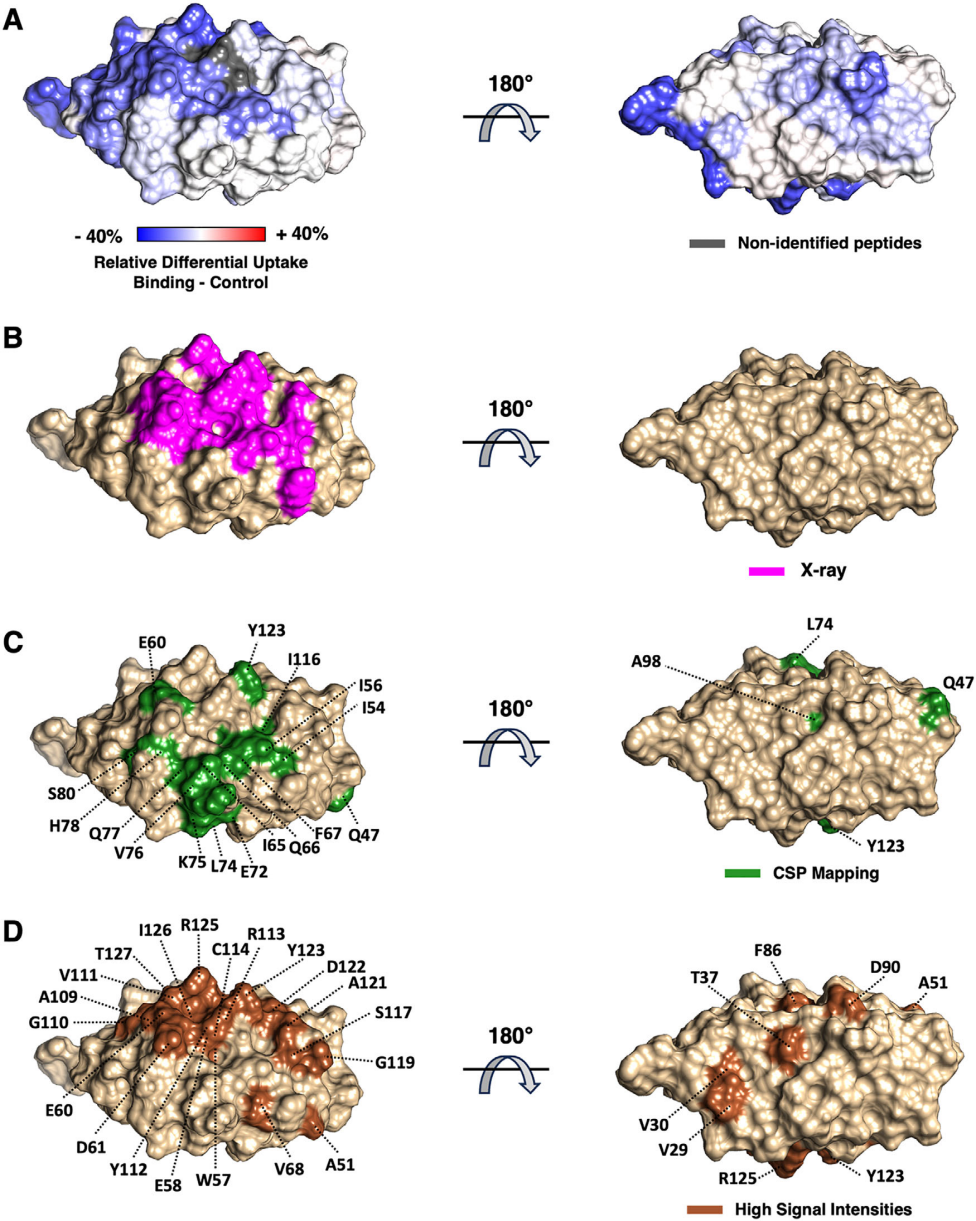
Mapping according to SSNMR results compared to HDX‐MS results and x‐ray data on crystal structure with PDB code: 5GRJ. The peptides experiencing significant differences in deuterium uptake according to HDX‐MS measurements are reported in the crystal structure of PD‐L1 (A). Deuterium uptake differences between binding and control conditions were measured after 60 min of D_2_O incubation and plotted onto the structure using a color gradient that ranges from blue (decreased) to white (unchanged) to red (increased) uptake. Non‐identified residues are shown in grey. In magenta are highlighted PD‐L1 residues identified by x‐ray crystallography to be involved in the interaction with Avelumab (B). Residues experiencing the largest differences in chemical shift (CSP) and strongest signal enhancement in SSNMR spectra of the Avelumab/PD‐L1 complex are highlighted in green (C) and brown (D), respectively, on the PD‐L1 crystal structure.

### 2.2 Epitope Mapping Using SSNMR

The binding of Avelumab to PD‐L1 was investigated using SSNMR, following the same procedure previously reported for anti‐PD‐L1 full‐length fusion protein [10]. ^13^C‐ and ^1^H‐detected SSNMR spectra were recorded on samples of PD‐L1 in complex with Avelumab (see Supporting Information) and compared with those previously acquired on the complex of PD‐L1 with the fusion protein.

The spectra of PD‐L1 in complex with Avelumab show a higher signal‐to‐noise ratio compared to those recorded on the PD‐L1/fusion protein complex. The enhanced signal‐to‐noise ratio allowed for the acquisition of additional 3D SSNMR spectra, thereby improving the resonance assignment of PD‐L1 interacting with this mAb. The new spectra enabled the assignment of additional residues not identified or present in the spectra of PD‐L1 when complexed with the fusion protein, though some of them still exhibited low intensities (Figure 5). Specifically, for the ^13^C‐detected spectra, the new assigned residues include K25, L48, Q66, L74, K75, V76, M115, and G120. For the ^1^H‐detected spectra, the additional residues are K41, K46, M59, E60, D61, Q66, L74, K75, V76, and L106. Only the signals of a few residues are still missing in the spectra of PD‐L1 in complex with Avelumab (H69, G70, Q83, A132‐Y134 for the ^13^C‐detected spectra; G70, Q83, Y134 for the ^1^H‐detected spectra). Summarizing, a total of 107 and 109 spin systems were assigned in the ^13^C‐ and ^1^H‐detected spectra, respectively, thus improving the previously reported assignment of 99 spin systems, available for PD‐L1 in complex with the fusion protein at the solid‐state. Importantly, the spectra of PD‐L1 in complex with Avelumab and the ones of PD‐L1 in complex with the full‐length anti‐PD‐L1 fusion protein are highly superimposable (Figure S7). These results further confirm that the Fab portion of Avelumab and the fusion protein share the same binding pose on the PD‐L1 target [10].

**FIGURE 5:**
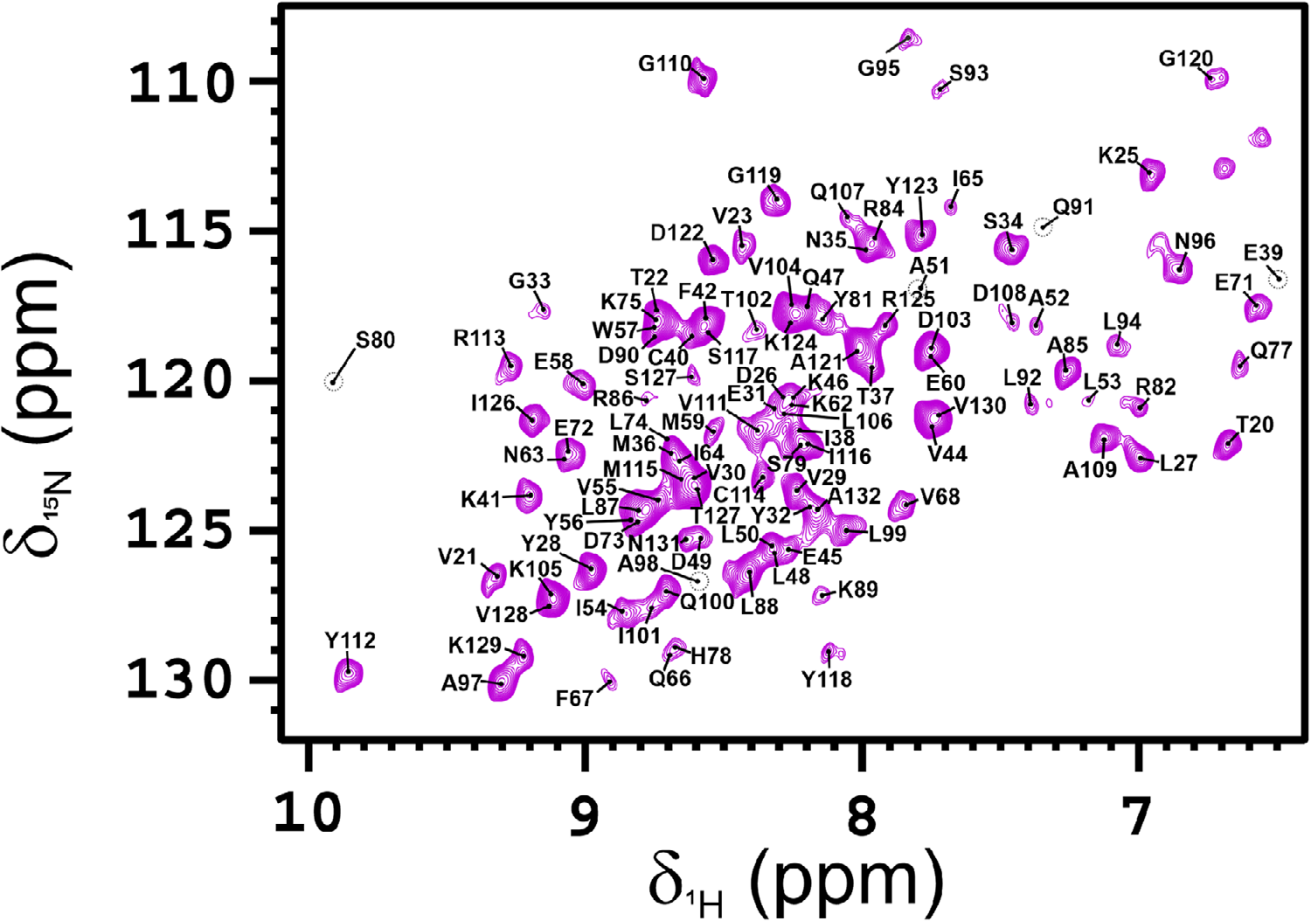
2D ^15^N ^1^H (H)NH CP of [U‐^2^H, ^13^C, ^15^N] PD‐L1 in complex with Avelumab. The spectrum was acquired on rehydrated freeze‐dried material, using a spectrometer operating at 800 MHz (^1^H Larmor frequency) with a MAS of 60 kHz and a temperature of ∼290 K. Assignments are reported on the signals.

The analysis of the chemical shift perturbation (CSP) of ^13^Cα/^15^N and ^1^H/^15^N signals of PD‐L1 in complex with Avelumab with respect to those of the free protein in solution is reported in Figures 4C and S8. The residues experiencing the largest CSPs (Q47, I54, Y56, E60, I65, Q66, F67, E72, L74, Q77, I116, Y123 according to ^13^Cα/^15^N chemical shift values; E60, I65, Q66, F67, L74, K75, V76, Q77, H78, S80, A98, I116 for the ^1^H/^15^N chemical shift values) are in the same region of PD‐L1 affected by the binding of anti‐PD‐L1 fusion protein. Interestingly, a small number of residues experience a larger effect upon binding of Avelumab (specifically I54, Y56, Q66, F67, and L74 based on ^13^Cα/^15^N chemical shift values; E60, Q66, L74, and V76 according to ^1^H/^15^N chemical shift values). In contrast, some residues are more influenced by the binding of the fusion protein (specifically E58, H78, Q83, C114, Y118 based on ^13^Cα/^15^N chemical shift values; M36, C40, V44, I64, V68, D108, G110, C114, Y118, D122, R125 and I126 according to ^1^H/^15^N chemical shift values). The residues that show the largest CSP in the complex between PD‐L1 and Avelumab identify a more clearly defined binding region on the PD‐L1 structure compared to the area previously identified by analyzing its complex with the fusion protein (see Figure S9). Specifically, in the presence of Avelumab, the β‐strand from I64 to F67 (C' β‐strand, Figure 1) and the loop from E72 to S80 (C''D loop, Figure 1) are affected by a larger CSP with respect to the rest of the protein sequence.

The intensity of the signals in the SSNMR spectra strictly depends on the related line broadening. Therefore, analyzing the signal intensity can yield insights into the local conformational heterogeneity of a specific region within the investigated protein. Indeed, the stabilization of the PD‐L1 structure into a unique conformation upon binding of Avelumab is responsible for the increased intensity of the signals corresponding to residues of the epitope mostly affected by the binding. Thus, the analysis of signal intensity can complement the CSP analysis to evaluate the effects of the binding. A qualitative analysis of the signal intensities of the 2D ^15^N ^1^H (H)NH CP correlation spectrum provides a clear signature of Avelumab binding pose. In particular, some signals, among those in non‐overlapping regions, are characterized by low intensities (i.e., G33, E39, A51, A52, L53, S80, R86, K89, Q91, L92, T102, Q107, D108). Interestingly, these signals belong to residues located on the surface of PD‐L1 opposite to the β‐platform known to bind Avelumab. Conversely, signals assigned to residues placed on the binding interface of PD‐L1 feature high intensities (E58, V68, A109, G110, V111, Y112, R113, C114, G119, D122, Y123, I126, Figure 5); of note are residues in the F β‐strand (from A109 to C114). The intensity of the signals in the 2D ^1^H‐^13^C projection of the 3D hCαNH spectrum was also analyzed, and the residues showing the highest signal intensity are also displayed in Figure 4D (V29, V30, T37, C40, A51, W57, E60, D61, R86, D90, G110, S117, G119, A121, D122, R125, T127, V128). This analysis further supports our findings, showing that signals assigned to the interaction interface (C, F, and G strands and the CC′′ loop) display high intensities. Interestingly, residues on the opposite face of PD‐L1 also display high intensities, suggesting that the stabilizing effect of Avelumab binding extends beyond the interaction interface.

### 2.3 Epitope Mapping Using HDX‐MS

HDX experiments were performed on free PD‐L1 and on its complex with Avelumab at a 1:2 molar ratio over the same time points used to determine the paratope mapping. The sequence coverage of PD‐L1 was 96% (62 peptides) with a redundancy of 4.88. (Figures S10 and S11).

Overall, the HDX results on PD‐L1 show a very pronounced decrease in deuterium uptake (down to −43%) located in close proximity to the potential binding site (e.g., I54‐E60 and D61‐K75), determined using x‐ray crystallography (Figure 4). A minor yet statistically significant increase in deuterium uptake (approximately 2%) was observed in the N‐terminal loop of the protein (F18‐Y28). This region, located on the opposite side of the interacting surface, acquires more flexibility upon interaction with Avelumab. The HDX‐MS data are consistent with the SSNMR results and align with the interacting surface previously observed in the x‐ray structure described by Liu et al. [8].

Surprisingly, additional PD‐L1 residues, previously not identified, exhibited significantly decreased uptake in the presence of Avelumab. Specifically, residues K124‐Y134, located at the C‐terminus, of the PD‐L1 construct display strong protection from deuterium exchange (Figures 3 and 4A). This protection may be due to a potential interaction of the C‐terminus with residues of the Avelumab light chain, which was not observed in the x‐ray structure (5GRJ) [8].

### 2.4 Analysis of the Integrated Data

Here, we integrated newly acquired HDX‐MS and NMR data with previously described x‐ray data that revealed novel features of PD‐L1 binding to Avelumab. A preliminary comparative analysis of the SSNMR data recorded on Avelumab with those previously collected on the fusion protein has revealed important findings. In particular, the PD‐L1 epitope identified by both HDX‐MS and SSNMR in complex with Avelumab closely overlaps with those identified for the fusion protein. The good match of data indicates that the sites selected for the covalent attachment of the extracellular domain proteins to Avelumab to produce the fusion protein, as well as the linker length, are sufficiently optimized not to interfere with the binding to PD‐L1. Conversely, the rearrangements that occur on the constant heavy 1 (C_H_1) domain loop of Avelumab in the presence of PD‐L1, as revealed by paratope mapping, indicate that any modifications in this region should be approached with caution, given the potential impact on complex formation.

A comparative analysis of signal intensities in the SSNMR spectra with differential deuterium uptake data from HDX‐MS experiments on PD‐L1 is shown in Figure 6.

**FIGURE 6:**
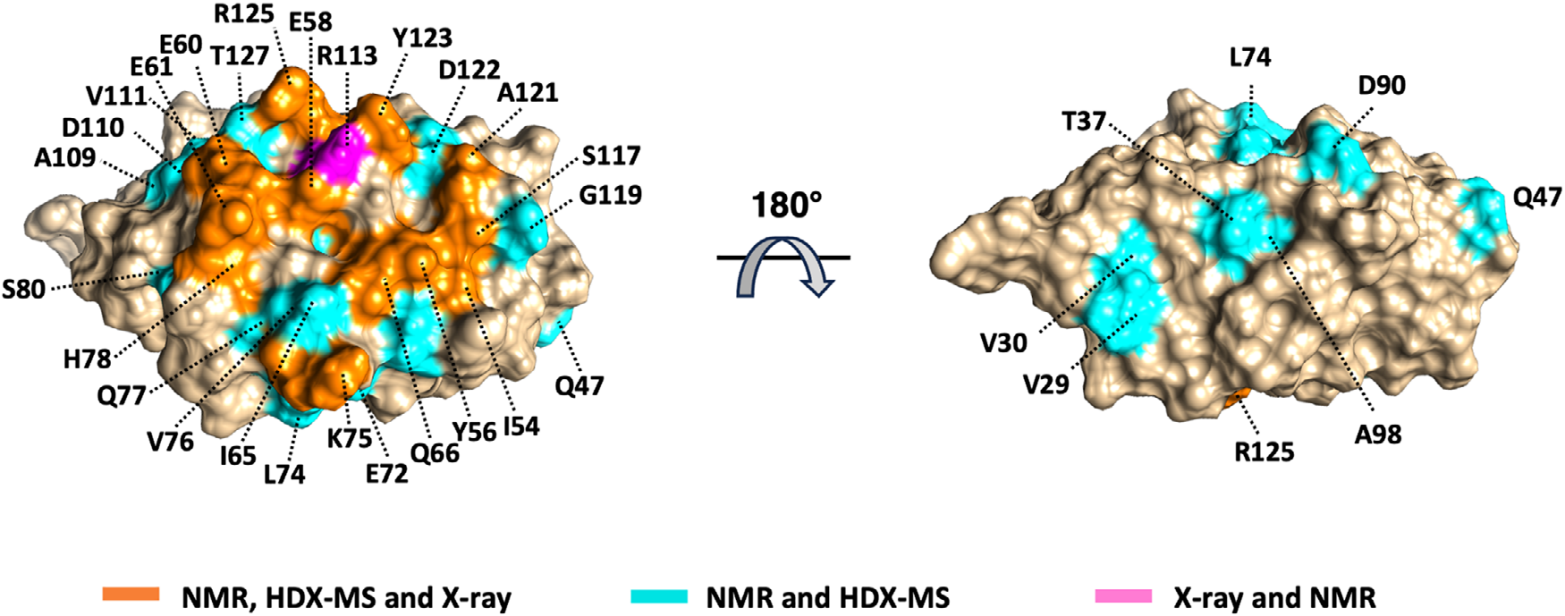
Structure of PD‐L1 shown on the surface, highlighting the residues involved in binding to Avelumab, as identified by a combination of x‐ray, NMR, and HDX‐MS data, with colors marked accordingly.

Regarding the HDX‐MS data, increased structural stabilization in, for example, more rigid secondary structure elements can also contribute to higher deuterium‐exchange protection. Upon Avelumab binding, significant HDX protection is observed for most residues in the β‐sheet, particularly in C, C′, and G β‐strands and on the EF loop, which directly interact with the antibody. Additional protection is detected in the β‐strand (D) and the short helix on the opposite face of the protein. However, the x‐ray data demonstrate that only a subset of the residues experiencing high HDX protection shows reduced solvent accessibility. This finding supports the notion of increased structural stabilization upon binding, which seems to contribute significantly to the observed high HDX protection. Regarding SSNMR data, both reduced conformational heterogeneity and motion contribute to a narrow linewidth and consequent high intensity of some signals in the spectra of the complex. The residues that show the most significant increase in intensity are primarily found in the β‐strands (C, F, G, and CC′ loop) directly involved in the interaction with Avelumab, but are also present in the β‐strands (A, B, and C′′) and the short helix located on the opposite side of the protein. Both methodologies agree in identifying the protein regions that experience a reduction in mobility and conformational heterogeneity upon complex formation. This is particularly relevant considering several studies indicating that the increase in binding affinity during antibody maturation is associated with enhanced rigidity and reduced conformational plasticity of the complementarity‐determining regions (CDRs) [74, 75]. This leads to the stabilization of a binding‐competent conformation rather than enhancing affinity primarily through the formation of new interactions by mutation‐acquired residues. In this context, the ability to monitor changes in conformational heterogeneity of single residues and peptides within the complex may provide a valuable proxy for tracking variations in binding affinity. Accordingly, the integrated use of these complementary techniques offers a new, powerful tool for gaining deeper insight into the structural consequences of protein–protein interactions, with potential applications in guiding antibody reshaping and evaluating its outcomes.

The HDX‐MS analysis indicates a potential interaction between the C‐terminus of the N‐terminal domain of PD‐L1 (K124‐Y134) and residues in the light chain of Avelumab (S21‐D28) that had not been previously identified by other biophysical techniques. Consequently, docking studies were conducted to investigate the possible structural basis for the decreased deuterium uptake observed at the C‐terminus of this PD‐L1 construct. The most populated cluster of PD‐L1/Avelumab complexes, obtained from the calculations, showed the lowest Haddock scores (Table S4) and displayed the same binding pose of Avelumab on the tandem domain of PD‐L1 as observed in the x‐ray structure (5GRJ). In the calculated structural model, the C‐terminus of the N‐terminal domain of PD‐L1 is bent so as to interact with residues of the light chain of Avelumab (G30, G31, Y32, N33, and Y34). In addition, this region is stabilized into a more rigid conformation by aromatic stacking interactions between the rings of two tyrosine residues at the binding interface, namely Y134 of PDL1 and Y32 of Avelumab (see Figure 7). This docking model offers a potential explanation for the decreased deuterium uptake at the C‐terminus of this PD‐L1 construct observed in the HDX‐MS experiments. It suggests that this region experiences reduced dynamics when the mAb is present, as it interacts with the G30‐Y34 segment of the light chain of Avelumab. This interaction could also account for the solvent protection observed for residues S21‐D28 in the light chain of the antibody. This may be due to allosteric structural stabilization occurring in this region when the C‐terminus of the PD‐L1 construct binds to nearby residues. However, the interaction suggested by HDX‐MS between the C‐terminus of the N‐terminal domain of PD‐L1 and the light chain of Avelumab is not supported by either the x‐ray or solid‐state NMR data. In the case of the x‐ray structure (5GRJ), the interaction between the C‐terminus and the light chain of Avelumab is likely not detected due to the crystallization of the full ectodomain of PD‐L1, where the membrane‐distal V‐set domain (residues A18–Y134) is connected to the membrane‐proximal C‐set domain (residues N135–H233). Therefore, in the tandem domain observed in the x‐ray structure, the C‐terminus of the V‐set domain forms the linker that connects the two domains and is also part of the C‐set domain itself. As a result, this region cannot move freely as it does in the construct used in the MS experiments. Additionally, the analysis of the solid‐state NMR spectra recorded on the complex involving the same PD‐L1 construct used for the MS experiments does not provide evidence for the aforementioned interaction, as the resonances of the residues at the C‐terminus are missing. The discrepancy between the NMR and MS findings is likely due to the transient nature of this interaction, which inhibits its observation by solid‐state NMR. Therefore, it is probable that this transient interaction does not occur in vivo. It may merely be an artifact related to the use of the isolated V‐set domain, which has a very flexible C‐terminus. In summary, while the existence and relevance of this interaction remain hypothetical, these findings highlight the importance of integrating different biophysical methods whose distinct sensitivity can also reveal potential transient interactions or artifacts related to experimental design and conditions.

**FIGURE 7:**
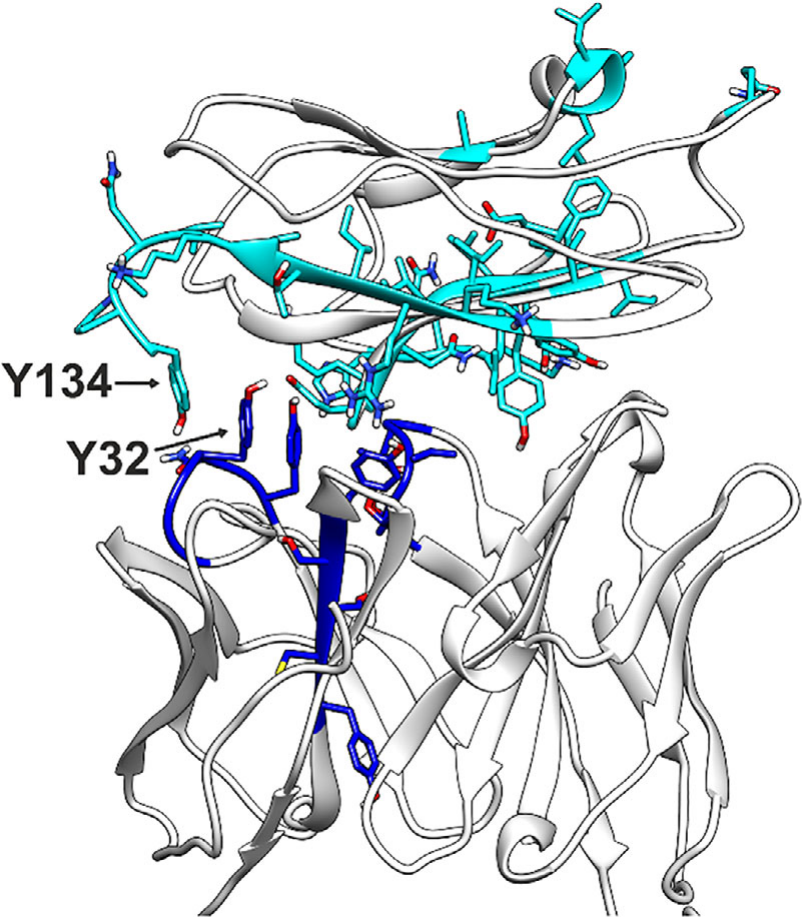
Structural model of the complex with the lowest energy between Avelumab and PD‐L1, generated by Haddock 2.4 calculation. The active residues used in the calculation are colored in cyan and blue for PD‐L1 and Avelumab, respectively. Tyrosine residues at the binding interface involved in stacking interactions are labeled in the figure.

## 3 Conclusion

Structural information is essential for the rational development of next‐generation antibody therapeutics, enabling the design of higher‐affinity binders while improving safety profiles by reducing immunogenicity. With the rise of biosimilar products, these studies have become crucial to support biosimilar development and, on the other hand, to defend intellectual property and patents. In this study, we show that the integration of SSNMR and HDX‐MS provides a robust and complementary framework for the structural characterization of mAb–antigen complexes in a formulation‐relevant context. SSNMR provides detailed insights into individual amino acids, down to the specific atoms involved in interactions, thereby defining highly accurate constraints for docking calculations and enabling the rational design of mutations to reshape binding properties. Complementing this structural resolution, HDX‐MS provides insights into both the epitope and paratope binding mechanisms. Applied to the Avelumab/PD‐L1 system, this approach enabled the definition of both the PD‐L1 epitope and the Avelumab paratope, while also revealing binding‐induced changes in structure and conformational dynamics in both partners. The strong agreement between SSNMR, HDX‐MS, and previously reported x‐ray data supports the reliability of the interaction model and confirms that Avelumab and the related fusion protein engage PD‐L1 through the same binding interface. At the same time, the additional protection observed at the C‐terminus of the isolated PD‐L1 construct highlights the sensitivity of HDX‐MS to transient or long‐range effects that may not be captured by other structural techniques and may also reflect construct‐specific behavior. Overall, this study illustrates how orthogonal biophysical methods can provide a more complete picture of antibody–antigen recognition than any single technique alone. More broadly, this workflow may be valuable for the characterization and optimization of therapeutic antibodies and other biologics, supporting rational engineering efforts in biotherapeutic development.

## Author Contributions

**Francesca Sacco**: methodology, investigation, data curation, formal analysis, writing – original draft. **Fabio Baroni**: conceptualization, validation, supervision, writing – original draft, investigation. **Angela Capolupo**: methodology, validation. **Sofia Petrocchi**: validation. **Jonathan Zöller**: methodology, investigation, formal analysis. **Linda Cerofolini**: investigation, methodology, validation, formal analysis, data curation, writing – original draft, visualization. **Pasquale Russomanno**: investigation, methodology, data curation. **Rebecca Calamandrei**: investigation. **Bianca Susini**: investigation. **Siyu Lin**: investigation. **Fabio D'amici**: validation. **Enrico Ravera**: supervision, validation, visualization. **Julian Langer**: conceptualization, investigation, writing – original draft, validation, supervision, resources. **Marco Fragai**: conceptualization, investigation, writing – original draft, validation, supervision, project administration, resources, funding acquisition.

## Conflicts of Interest

Francesca Sacco, Fabio Baroni, Angela Capolupo, Sofia Petrocchi and Fabio d′Amici were employees of Merck Serono S.p.a, Guidonia, RM, Italy; an affiliate of Merck KGaA at the date of the analyses. This research was performed using as case study sample a product in development by Merck KGaA. While Merck KGaA has filed for patent protection regarding the product in development, the technology described in this manuscript is independent from this product of Merck KGaA. No patents or patent applications have been filed for the technology described in this manuscript

## Supporting information



Experimental Details are reported in the Supporting Information. The authors have cited additional references within the Supporting Information [54, 76, 77, 78, 79, 80, 81, 82, 83, 84, 85, 86, 87, 88, 89, 90].
**Supporting File**: anie74307‐sup‐0001‐SuppMat.docx.

## Data Availability

The data that support the findings of this study are available in the Supporting Information of this article
