# Supplementary material for: Characterization of Full‐Length Antibody Complexes Through HDX‐Mass Spectrometry Coupled With NMR Spectroscopy: New Insight Into Avelumab/PD‐L1 Interaction
Source: Angew Chem Int Ed Engl. 2026 Aug 22;65(42):e1540355. doi: 10.1002/anie.1540355 (PMC13653614; doi:10.1002/anie.1540355)
Supplement: Supplementary file 1 [file ANIE-65-e1540355-s001.docx]

**Supporting Information**

**Characterization of Full-Length Antibody Complexes through HDX-Mass Spectrometry coupled with NMR Spectroscopy: New Insight into Avelumab/PD-L1 Interaction**

Francesca Sacco ^[a][b]^; Fabio Baroni^[b]^; Angela Capolupo^[b]^; Sofia Petrocchi^[b]^; Jonathan Zöller^[c]^; Linda Cerofolini^[a][d]^; Pasquale Russomanno^[a][d]^; Rebecca Calamandrei^[a][d]^; Bianca Susini^[a][d]^_,_ Siyu Lin^[a][d]^, Fabio D’Amici^[b]^; Enrico Ravera^[a][d]^; Julian Langer ^[c][e]*^, Marco Fragai^[a][d]*^

[a]	Dr. F. Sacco, Dr. L. Cerofolini, Dr. P. Russomanno, R. Calamandrei, B. Susini, S. Lin, Prof. E. Ravera, Prof. M. Fragai
CERM-CIRMMP
University of Florence
Via Luigi Sacconi 6, 50019, Sesto Fiorentino, Italy
E-mail: marco.fragai@unifi.it

[b]	Dr. F. Sacco, Dr. Fabio Baroni, Dr. Angela Capolupo, Dr. Sofia Petrocchi, Dr. Fabio D’Amici,
Analytical Development & Innovation
NBE, EMD Serono Inc
Guidonia (Rome), Italy

[c]	J. Zöller, Prof. J. Langer
Max Planck Institute of Biophysics
Frankfurt am Main, Germany
E-mail: [julian.langer@biophys.mpg.de](mailto:julian.langer@biophys.mpg.de)

[d] Dr. L. Cerofolini, Dr. P. Russomanno, R. Calamandrei, B. Susini, S. Lin, Prof. E. Ravera, Prof. M. Fragai
Department of Chemistry (DICUS)
University of Florence
Via della Lastruccia 3, 50019, Sesto Fiorentino, Italy

[e]	Prof. J. Langer
Max Planck Institute for Brain Research
Frankfurt am Main, Germany.

**Table of content**

Experimental Methods	Page S3

Supporting Results	Page S6

Figure S1.	Page S6

Table S1	Page S7

Figure S2.	Page S7

Figure S3.	Page S8

Figure S4	Page S9

Figure S5.	Page S10

Figure S6	Page S11

Figure S7	Page S12

Figure S8.	Page S13

Figure S9.	Page S14

Figure S10.	Page S15

Figure S11	Page S15

Table S2	Page S16

Table S3	Page S17

Table S4	Page S18

**EXPERIMENTAL METHODS**

**Expression and Purification of PD-L1 Ectodomain:**

Escherichia coli BL21 (DE3) cells were transformed with pET-21a (+) plasmid encoding PD-L1 (residues A18-Y134;- AFTVTVPKDLYVVEYGSNMTIECKFPVEKQLDLAALIVYWEMEDKNIIQFVHGEEDLKVQHSS

YRQRARLLKDQLSLGNAALQITDVKLQDAGVYRCMISYGGADYKRITVKVNAPY). Cells were cultured in LB medium supplied with 1 mL of a 0.1 mg/mL solution of ampicillin, grown at 310 K until OD_600_ reached 0.6/0.8, and then induced with 1 mmol·dm^–3^ isopropyl β-D-1-thiogalactopyranoside. They were further grown at 310 K overnight and then harvested by centrifugation at 6500 rpm (JA-10 Beckman Coulter) for 15 min at 277 K. Pellet was suspended, at first, in 50 mmol·dm^–3^ Tris-HCl pH 8.0, 200  mmol·dm^–3^ NaCl, 10  mmol·dm^–3^ β-mercaptoethanol, and 10  mmol·dm^–3^ EDTA, (50 mL per liter of culture) and sonicated for 30 seconds 10 times on ice at 277 K. The suspension was centrifuged at 40,000 rpm (F15-6x100y Thermo Scientific) for 40 min, and the supernatant was discarded. The recovered pellet was resuspended in 50 mmol·dm^–3^ Tris-HCl pH 8.0, 200  mmol·dm^–3^ NaCl, 10  mmol·dm^–3^ β-mercaptoethanol, and guanidinium chloride (25 mL per liter of culture) and again incubated at 277 K overnight under magnetic stirring. The resulting suspension was centrifuged at 40,000 rpm (F15-6x100y Thermo Scientific) for 40 min. The pellet was discarded, whereas the supernatant containing the denatured protein solution was diluted in a refolding buffer containing 0.1 M Tris-HCl, pH 8.5, 1 M arginine, 0.25 mmol·dm^–3^ reduced glutathione, and 0.25 mmol·dm^–3^ oxidized glutathione. The solution was incubated at 277 K under stirring, for 12-18 h, cleared by passing a 0.45-μm filter, and then dialyzed extensively against 50 mmol·dm^–3^ Tris, pH 8.0, 150 mmol·dm^–3^ NaCl. The protein solution was concentrated with an Amicon® Stirred Cell and then purified by size exclusion chromatography on HiLoad Superdex 26/60 75 pg (GE Healthcare) previously equilibrated in 0.1 mmol·dm^–3^ HEPES pH 6.8 and 20 mmol·dm^–3^ NaCl.

**Preparation of the complex Avelumab:PD-L1 for HDX-MS studies**

Desalting-removal columns (Zeba Spin Desalting Columns, 0.5 mL, Thermo Scientific) were used to reduce the amount of Tween 20, present in the pharmaceutical antibody formulation (10 mmol·dm^–3^ Sodium acetate, 51 mg/mL d-Mannitol, 0.5 mg/mL Tween 20, pH 5.2), and to exchange the buffer to the equilibration buffer needed for HDX-MS analysis (20 mmol·dm^–3^ Tris HCl, 50 mmol·dm^–3^ NaCl at pH 7.5). In the binding condition, we prepared an Avelumab:PD-L1 complex by mixing Avelumab and PD-L1, both in the equilibration buffer, to a final concentration of 15 and 30 µmol·dm^–3^, respectively. In the control condition, we used Avelumab without the ligand.

Surface plasmon resonance has been performed to confirm the binding event in the chosen experimental conditions (Figure S1, Table S1).

**HDX-MS data acquisition and processing**

HDX-MS experiments have been performed and interpreted according to the community-based recommendations.^[49]^ Both the antibody (control) and the complex (binding) samples were diluted 15-fold at 20°C with the deuterated labeling buffer (20 mmol·dm^–3^ Tris HCl, 50 mmol·dm^–3^ NaCl pD=7.5) and quenched 1:1 to a final pH of 2.5 at 0°C after labeling for 0.5, 3, 15, and 60 min (quenching buffer: 150 mmol·dm^–3^ KPi, 6 mol·dm^–3^ Urea, 200 mmol·dm^–3^ TCEP, pH 2.20), on an HDX PAL robot (LEAP Technologies). Unlabeled reference samples were measured for both binding and control samples in the same conditions as the labeled ones, by diluting the samples in equilibrium buffer (20 mmol·dm^–3^ Tris HCl, 50 mmol·dm^–3^ NaCl pH 7.5) at 20°C. Each time point was measured in quadruplicates. Peptides were generated by online digestion performed at 20 °C with a constant flow rate of 100 μL/min, using a Pepsin/Nepenthensin-2 column (AffiPro) and desalted on a C18 column (ACQUITY UPLC BEH C18 1.7 μm VanGuard column, Waters) for 3 min with solvent A (0.23% formic acid in MS-grade water, pH 2.5). Subsequently, the peptide mixture was separated on a reverse phase C18 analytical column (ACQUITY UPLC BEH C18 1.7 μm, 1 × 100 mm column, Waters) using an 8 min linear gradient (with 7 min of washing and 2 min of equilibration) at a 40 μL/min flow rate with increasing solvent B (0.23% formic acid in acetonitrile) concentrations (5%–40% (v/v)). Eluting peptides were measured on a Synapt G2-Si mass spectrometer with an electrospray ionization source operated in a positive ionization mode. We utilized ion mobility for additional peptide separation and identification (RT, *m/z*, drift time). For lock-mass calibration, human Glu-Fibrinopeptide B was recorded throughout the analysis to ensure mass accuracy. Peptides were identified from non-deuterated measurements using ProteinLynx Global Server 3.0.3. (PLGS, Waters) for each condition (control and binding). The manual evaluation of the mass spectra was performed using the software DynamX 3.0 (Waters) for all peptides identified in, at least, 6 out of 8 replicates. The PD-L1 epitope mapping was performed using the same protocol as in the Avelumab experiment.

**Statistical analysis of HDX-MS data**

After the manual curation of all MS spectra in DynamX (Waters), all peptides were subjected to a two-stage t-test based on Houde et al. ^[50]^ to analyze the statistical significance in deuterium uptake differences. The statistical analysis was performed with an in-house written R-script previously described by Demmer et al.^[51]^ The standard error of the mean (SEM) of each peptide and time point was used to test for significance in the first stage (P=0.05). Peptides displaying significance at least three time points were submitted to the second stage, in which the sum of the SEM for each peptide is evaluated (P=0.01). Only peptides passing both steps were used for further evaluation and the creation of the deuterium uptake difference maps.

**SSNMR measurements**

Samples of complexes of [U-^13^C, ^15^N] and [U-^2^H, ^13^C, ^15^N] PD-L1 with Avelumab for SSNMR were prepared by adding increasing aliquots of the pharmaceutical formulation of the mAb [20 mg·cm^-3^ (~ 139 µmol·dm^-3^)] to solutions of PD-L1 [~ 45 µmol·dm^-3^ in 10 mmol·dm^-3^ Tris, 20 mmol·dm^-3^ NaCl, pH 8] and the formation of the complexes monitored by recording 2D ^1^H-^15^N SOFAST-HMQC solution NMR spectra.^[52]^ An amount of PD-L1 corresponding to ~ 50% was complexed with Avelumab, and the excess of unbound PD-L1 was then purified, and the buffer exchanged to 1 mmol·dm^-3^ HEPES (pH 6.8) and 4 mmol·dm^-3^ NaCl using a centrifugal concentrator and HiLoad Superdex 16/60 200pg gel-filtration chromatography. The solutions of the complexes were freeze-dried, and the materials used to pack 3.2- zirconia thick walls and 1.3-mm rotors (~ 23 mg and 3 mg of material, respectively). The dry samples were then rehydrated with MilliQ H_2_O as previously described. ^[9]^Silicon plugs (courtesy of Bruker Biospin) placed below the turbine cap were used to close the 3.2 mm rotors and preserve hydration.

The SSNMR spectra of PD-L1 in the presence of Avelumab were collected on a Bruker Avance III spectrometer operating at 800 MHz, ^1^H Larmor frequency (18.8 T, 201.2 MHz ^13^C Larmor frequency), equipped with a Bruker 3.2 mm Efree and a Bruker 1.3 mm NCH probe-heads. The spectra were recorded at 14 and 60 kHz MAS frequencies, for the 3.2- and 1.3-mm rotors respectively, and the sample temperature was kept at ~ 290 K.

Standard ^13^C-detected SSNMR spectra [2D ^15^N-^13^C NCA, ^15^N-^13^C NCO, ^13^C-^13^C DARR, mixing time 50 ms, 3D NCACX and CANCO] ^[53–56]^ were acquired on the sample in 3.2 mm rotor, while ^1^H-detected SSNMR spectra [2D ^15^N-^1^H (H)NH CP-HSQC, 3D (H)CANH, 3D (H)CONH and 3D (H)(CA)CB(CA)NH] were acquired on the sample in 1.3 mm rotor, using the pulse sequences reported in the literature.^[57–61]^ Experimental details are reported in Table S2 and S3.

All the spectra were processed with the Bruker TopSpin 3.7 software and analyzed with the program CARA.^[62]^

**Modeling of PD-L1/Avelumab complex**

The model of the complex between PD-L1 and Avelumab was obtained by performing docking calculations with the software HADDOCK 2.4^[63,64]^ on the web interface (<https://wenmr.science.uu.nl/haddock2.4>), using the *Guru* access. In the calculation, during the rigid-body docking, 1000 complexes were generated, then 200 structures were selected for the semi-flexible simulated annealing in torsion angle space, and finally refined in Cartesian space with explicit solvent. The model structure of Avelumab^[65]^ provided the input coordinates for the mAb, while the input coordinates for our PD-L1 construct (residues A18-Y134) were obtained from the PDB structure 5GRJ.^[8]^ The residues identified by HDX-MS experiments (for both Avelumab and PD-L1), with a decreased deuterium uptake lower than -20%, and by SSNMR (for PD-L1 only), with a chemical shift perturbation (CSP) larger than a selected threshold (mean + σ), were defined as *active* residues in the HADDOCK calculation, and used as ambiguous restraints [*active* residues for Avelumab: G30, G31, Y32, N33, Y34, Y89, C90, S91, S92 and Y93 from the light chain, and G102, T103, V104, T105 and T106 from the heavy chain; *active* residues for PD-L1: Q47, I54, Y56, E60, K62, N63, I64, I65, Q66, F67, E72, L74, K75, V76, Q77, H78, S80, L92, S93, L94, A98, I116, Y123, K124, R125, I126, T127, V128, K129, V130, N131, A132, P133, Y134]. The C-terminal segment of PD-L1 (residues: V130-Y134) was defined as semi- and fully- flexible during the simulated annealing in torsion angle space and the refinement in Cartesian space, respectively, to allow possible conformational changes driven by experimental restraints.

**Supporting Results**

**Surface Plasmon Resonance**

Surface Plasmon Resonance (SPR) has been applied to study the interaction between PD-L1 in solution and Avelumab, attached on a sensor chip surface. The sensor chip is a glass slide coated with a thin layer of gold, on which a matrix of carboxymethylated dextran is covalently attached. The gold is required for generation of the SPR response, as at the interface between media of different refractive index the SPR phenomenon occurs. In the Biacore system, media are the glass of the sensor chip and the sample solution; the conducting film is the gold layer on the sensor chip surface. Under conditions of total internal reflection, the light leaks an electric field (evanescent wave field) across the interface into the medium of lower refractive index. Because the evanescent wave field penetrates the solution, changes in solute concentration can be measured as changes in the SPR conditions.

SPR experiments were performed on a Biacore T200 instrument (Cytiva). Avelumab was captured via the Fc portion by a Protein A sensor chip (cat. 29127556; Cytiva). The mAb, diluted at 10 nM, was injected at 30 µL/min for 1 minute to obtain about 700 RU of capture level. A range of 5 PD-L1 concentrations (7.5, 15, 30, 60 and 120 nM) was flown over the chip in single cycle kinetic mode and the contact time was set at 3 minutes. At the end of the highest concentration’s injection the complex was left to dissociate for 90 minutes. The experiment was performed at 30 µL/min. Before PD-L1 injection, a blank cycle was run applying the same parameters set for the analyte (PD-L1), by injecting the running buffer (HBS-EP+, prepared from HBS-EP+ 10X cat. BR100826; Cytiva). At the end of each cycle the chip surface was regenerated by injection of 10 mM glycine-HCl pH 1.5 (cat. BR100354; Cytiva) at 30 µL/min for 30 seconds.

In Figure S1 and Table S1 are reported obtained results.

**Figure S1**. **SPR sensorgrams**. Sensorgram data were processed and fitted using the Biacore T200 Evaluation software (Version 3.1). The sensorgram (on the right) was produced plotting the binding response (resonance unit on the Y-axis) versus the time (seconds on the X-axis). On the left: sensorgram magnification of the association phase.

*Figure S SEQ Figure_S \* ARABIC 1. Sensorgram with zoom of the association part (on the left).*

**Table S1**. **SPR results (ka, kd and KD)**. Quantitative analysis of PD-L1 binding to Avelumab was performed applying a 1:1 binding model. The equilibrium dissociation constant (KD) was estimated together with association and dissociation rates (ka and kd, respectively). KD value was obtained from the average of 2 independent experimental runs. Due to the very slow complex dissociation, it was applied a long dissociation time to ensure enough signal decrease, necessary for the evaluation of the kd by the software.

**Table S1. Results**

| N=2 | ka (1/Ms) | kd (1/s) | KD (pM) |
| --- | --- | --- | --- |
| Average | 4,78E+04 | 2,18E-05 | 456 |
| CV % | 0,4 | 4,5 | 4,9 |

**Figure S2**. Sequence coverage of heavy chain (top) and light chain (bottom) of Avelumab (KEGG accession number D10817). Peptides generated through online digestion and analyzed by LC-MS/MS are represented as green bars along the sequence.

**Figure S3**. Heat maps of heavy (top) and light (bottom) chains. The differential deuterium uptake (binding minus control) is represented with a color gradient ranging from blue (decreased) to white (unchanged) to red (increased uptake), with a threshold of ± 20%.

**Figure S4**. Close up view of the HDX-MS results after 60 min of labeling obtained for the fragment antigen-binding (Fab) region of Avelumab. The Fab is depicted in complex with the PD-L1 ectodomain (PDB: 5GRJ). Deuterium uptake differences between binding and control are plotted on the structure with a color gradient as described in Figure 2. Deuterium uptake plots of peptides showing the most pronounced differences are reported as well (error bars indicate the standard deviation of the relative deuterium uptake for each time point). Representative peptide uptake plots are shown with standard deviation error bars. The control condition is shown in red, and the binding condition in blue. Statistical significance of differences in deuterium uptake was assessed using a two-sided t-test; all time points shown indicate statistically significant differences.

**Figure S5**. Close up view of the HDX-MS results obtained for the constant domains of heavy and light chains of Avelumab. Deuterium uptake differences between binding and control samples were determined after 60 minutes and are indicated with a color gradient ranging from blue (decreased) to white (unchanged) to red (increased uptake), with a threshold of ± 20%. Non-identified residues are colored in gray, and cysteine residues in yellow. Deuterium uptake plots are reported with error bars showing the standard deviation of the relative deuterium uptake for each time point*.* Representative peptide uptake plots are shown with standard deviation error bars. The control condition is shown in red, and the binding condition in blue. Statistical significance of differences in deuterium uptake was assessed using a two-sided t-test; all time points shown indicate statistically significant differences.

**Figure S6**. Close up view of the HDX-MS results obtained for the fragment crystallizable (Fc) of Avelumab. Deuterium uptake differences between binding and control samples are shown after 60 minutes and are indicated with the color gradient described in Figure S4. Deuterium uptake plots are reported with error bars showing the standard deviation of the relative deuterium uptake for each time point. Representative peptide uptake plots are shown with standard deviation error bars. The control condition is shown in red, and the binding condition in blue. Statistical significance of differences in deuterium uptake was assessed using a two-sided t-test; all time points shown indicate statistically significant differences.

**Figure S7**. 2D ^15^N^13^C NCA (top) and ^15^N ^1^H (H)NH CP (bottom) spectra of [U- ^13^C, ^15^N] and [U-^2^H, ^13^C, ^15^N] PD-L1 in complex with the anti-PD-L1 fusion protein (red) and Avelumab (blue). All the spectra were acquired on rehydrated freeze-dried materials, using a spectrometer operating at 800 MHz (^1^H Larmor frequency) with a MAS of 14 and 60 kHz used for the NCA and (H)NH spectra, respectively, and a temperature of ∼290 K.

**Figure S8**. Chemical shift perturbation (CSP) of free PD-L1 in solution with respect to the rehydrated freeze-dried complexes of PD-L1 with anti-PD-L1 fusion protein (A, B) and with Avelumab (C, D) in the solid state, evaluated according to the formula (A, C) $\Delta\delta= \frac{1}{2}\sqrt{{({{\Delta\delta}_{C\alpha}}/2)}^{2}+{({{\Delta\delta}_{N}}/5)}^{2}}$ and (B, D) $\Delta\delta= \frac{1}{2}\sqrt{{{\Delta\delta}_{H}}^{2}+{({{\Delta\delta}_{N}}/5)}^{2}}$. The residues experiencing the largest variations (> mean + σ) have been highlighted in magenta (A, C) and blue (B, D). The secondary structure representation is reported on the top of the plot. The β-strands facing Avelumab in the structure of the complex are highlighted in yellow.

**Figure S9**. Chemical shift perturbation mapping according to SSNMR results. Residues experiencing significant CSP according to the data recorded on PD-L1 by SSNMR in the presence of anti-PD-L1 fusion protein (blue) and Avelumab (green).

**Figure S10**. Sequence coverage of PD-L1 ectodomain. Peptides generated through online digestion and analyzed by LC-MS/MS are represented as green bars.

**Figure S11**. Heat maps of PD-L1 ectodomain. The differential deuterium uptake (binding minus control) is represented with a color gradient ranging from blue (decreased) to white (unchanged) to red (increased uptake), with a threshold of ± 20%.

**Table S2.** Parameters used for the acquisition of ^13^C-detected SSNMR spectra.

| **Experiment** | **2D NCA** | **2D NCO** | **2D DARR** | **3D NCACX** | **3D CANCO** |
| --- | --- | --- | --- | --- | --- |
| **TD points** | 2048 (f2) x 80 (f1) | 2048 (f2) x 80 (f1) | 2048 (f2) x 1024 (f1) | 2048 (f3) x 64 (f2) x 38 (f1) | 2048 (f3) x 38 (f2) x 64 (f1) |
| **Number of scans** | 1024 | 1024 | 128 | 144 | 64 |
| **D1 (sec)** | 1.5 | 1.5 | 1.5 | 1.5 | 1.5 |
| **Transfer 1** | HN CP | HN CP | HC CP | HN CP | HC CP |
| **field [kHz]** | 60(H)46(N) | 60(H)46(N) | 70(H)42(C) | 60(H)46(N) | 70(H)42(C) |
| **shape if applicable** | 100-70 ramp on H | 100-70 ramp on H | 100-70 ramp on H | 100-70 ramp on H | 100-70 ramp on H |
| **time [ms]** | 0.75 | 0.75 | 1 | 0.75 | 1 |
| **Transfer 2** | NCA DCP | NCO DCP | mixing | NCA DCP | CAN DCP |
| **field [kHz]** | 80(H)35(N)21(C) | 80(H)21(N)35(C) |  | 80(H)35(N)21(C) | 80(H)35(N)21(C) |
| **shape if applicable** | tangent on C | tangent on C |  | tangent on C | tangent on C |
| **time [ms]** | 4 | 5 | 50 | 4 | 4 |
| **Transfer 3** |  |  |  | mixing | NCO DCP |
| **field [kHz]** |  |  |  |  | 80(H)21(N)35(C) |
| **shape if applicable** |  |  |  |  | tangent on C |
| **time [ms]** |  |  |  | 15 | 5 |

**Table S3.** Parameters used for the acquisition of ^1^H-detected SSNMR spectra. The (H)(CA)CB(CA)NH experiment used two spin echoes for the out-and-back CA-CB transfer, with Q3 refocusing pulse selective for the entire ^13^C aliphatic region of length 150 μs, rf-field peak amplitude 25 kHz, and half-echo delays of 7.2 ms.

| **Experiment** | **2D (H)NH** | **3D (H)CONH** | **3D (H)CANH** | **2D ^1^H-^13^C plane (H)(CA)CB(CA)NH** |
| --- | --- | --- | --- | --- |
| **TD points** | 2048 (f2) x 128 (f1) | 2048 (f3) x 46 (f2) x 54 (f1) | 2048 (f3) x 48 (f2) x 64 (f1) | 1024 (f3) x 30 (f2) x 56 (f1) |
| **Number of scans** | 240 | 128 | 160 | 192 |
| **D1 (sec)** | 1.5 | 1.5 | 1.5 | 1.5 |
| **Transfer 1** | HN CP | HCO CP | HCA CP | HCA CP |
| **field [kHz]** | 50(H)10(N) | 50(H)10(C) | 50(H)10(C) | 50(H)10(C) |
| **shape if applicable** | 70-100 ramp on H | 70-100 ramp on H | 70-100 ramp on H | 70-100 ramp on H |
| **time [ms]** | 2 | 5 | 4 | 4 |
| **Transfer 2** | NH CP | CON CP | CAN CP | CACB out and back |
| **field [kHz]** | 50(H)10(N) | 35(C)25(N) | 35(C)25(N) |  |
| **shape if applicable** | 100-70 ramp on H | tangent on N | tangent on N |  |
| **time [ms]** | 0.4 | 7 | 8 |  |
| **Transfer 3** |  | NH CP | NH CP | CAN CP |
| **field [kHz]** |  | 50(H)10(N) | 50(H)10(N) | 35(C)25(N) |
| **shape if applicable** |  | 100-70 ramp on H | 100-70 ramp on H | tangent on N |
| **time [ms]** |  | 0.4 | 0.4 | 8 |
| **Transfer 4** |  |  |  | NH CP |
| **field [kHz]** |  |  |  | 50(H)10(N) |
| **shape if applicable** |  |  |  | 100-70 ramp on H |
| **time [ms]** |  |  |  | 0.4 |

**Table S4.** HADDOCK statistics evaluated on the 200 water refined models of PD-L1 in complex with Avelumab. The reported data are related to the best 4 structural models of the main clusters in agreement with the experimental data with the lowest HADDOCK-scores.

|  | **Clust I** | **Clust II** | **Clust III** | **Clust IV** | **Clust V** |
| --- | --- | --- | --- | --- | --- |
| **HADDOCK-score** | -92 ± 4 | -71 ± 7 | -41 ± 2 | -44 ± 3 | -52 ± 5 |
| **Nstrc** | 43 | 14 | 12 | 11 | 9 |
| **RMSD** | 0.4 ± 0.2 | 0.4 ±0.2 | 0.6 ± 0.4 | 0.8 ± 0.6 | 0.3 ± 0.2 |
| **Edesolv** | -18 ± 2 | -8 ± 1 | -1 ± 1 | -12 ± 3 | -7 ± 3 |
| **BSA** | 1861 ± 19 | 1506 ± 37 | 1083 ± 33 | 1358 ± 56 | 1410 ± 43 |
| **Eair** | 333 ± 29 | 271 ± 63 | 228 ± 63 | 291 ± 49 | 265 ± 26 |
